# Pseudo-Subarachnoid Hemorrhage After Spinal Myelography Mimicking Acute Subarachnoid Hemorrhage

**DOI:** 10.7759/cureus.106000

**Published:** 2026-03-27

**Authors:** Ryosuke Doi, Tatsuya Tanaka, Ren Fujiwara, Akira Matsuno

**Affiliations:** 1 Department of Neurosurgery, International University of Health and Welfare, Narita Hospital, Narita, JPN; 2 Department of Neurosurgery, Narita-Tomisato Tokushukai Hospital, Chiba, JPN

**Keywords:** computed tomography, contrast media, diagnostic pitfall, myelography, pseudo-subarachnoid hemorrhage, subarachnoid hemorrhage mimic

## Abstract

Subarachnoid hemorrhage (SAH) is a life-threatening condition typically diagnosed on non-contrast computed tomography (CT) by the presence of hyperdense blood within the subarachnoid spaces. However, several non-hemorrhagic conditions can mimic this appearance, a phenomenon known as pseudo-subarachnoid hemorrhage (pseudo-SAH). Among these, contrast-related pseudo-SAH has been increasingly recognized following diagnostic and therapeutic procedures.

We report a case of pseudo-SAH caused by migration of intrathecal contrast medium after spinal myelography that initially mimicked spontaneous SAH on CT. Head CT demonstrated diffuse high attenuation within the subarachnoid spaces, with attenuation values higher than typically observed in true SAH. Follow-up CT revealed rapid resolution of the hyperdense areas. Based on these findings and the recent history of myelography, a diagnosis of contrast-related pseudo-SAH was made.

This case highlights that careful evaluation of clinical history, CT attenuation values, and temporal changes in imaging findings is essential for differentiating pseudo-SAH from true SAH. Awareness of this entity may help avoid unnecessary invasive investigations and inappropriate management.

## Introduction

Subarachnoid hemorrhage (SAH) is a life-threatening neurological condition most commonly caused by rupture of an intracranial aneurysm and is typically diagnosed on non-contrast head computed tomography (CT) by the presence of hyperdense blood within the subarachnoid spaces, including the basal cisterns and Sylvian fissures. Rapid and accurate diagnosis is essential because SAH requires urgent evaluation and treatment.

However, several non-hemorrhagic conditions can produce CT findings that mimic SAH. This phenomenon, known as pseudo-subarachnoid hemorrhage (pseudo-SAH), is characterized by increased attenuation within the subarachnoid spaces without true bleeding. Pseudo-SAH has been reported in various clinical settings, including diffuse cerebral edema associated with hypoxic-ischemic encephalopathy, severe brain swelling, chronic subdural hematoma, and metabolic disturbances such as water intoxication [1-4]. More recently, pseudo-SAH has also been described following intrathecal or intravascular contrast administration during diagnostic or therapeutic procedures [5-7].

 Intrathecal contrast migration following spinal procedures represents a rare but clinically important mechanism of pseudo-SAH that can closely mimic true subarachnoid hemorrhage on CT. Differentiating pseudo-SAH from true SAH is clinically important because misinterpretation may lead to unnecessary invasive diagnostic procedures or inappropriate management, particularly in emergency settings. Careful evaluation of clinical history, CT attenuation values, and temporal changes in imaging findings is therefore essential for accurate diagnosis.

Here, we report a case of pseudo-SAH caused by migration of intrathecal contrast medium following spinal myelography that initially mimicked spontaneous SAH on CT.

## Case presentation

A patient with recurrent lumbar spinal canal stenosis was scheduled to undergo reoperation at a local hospital. In the months preceding presentation, the patient had experienced progressive gait disturbance and gradual deterioration in activities of daily living, with repeated minor falls at home, which were considered attributable to the worsening lumbar spinal canal stenosis. As part of the preoperative assessment, the patient was referred to the department of neurology to evaluate for any intracranial pathology that might contribute to the gait disturbance or affect surgical planning. At that visit, a screening non-contrast head CT was obtained. Although the patient had no symptoms suggestive of acute subarachnoid hemorrhage, such as sudden severe headache, vomiting, loss of consciousness, or acute focal neurological deficits, the CT demonstrated hyperdense areas within the subarachnoid spaces, raising suspicion for SAH. The patient was therefore referred to our hospital for further evaluation.

The patient had a history of lumbar spinal canal stenosis, cerebral infarction, chronic obstructive pulmonary disease, hypertension, and dyslipidemia. Medications included aspirin, rosuvastatin, and other routine drugs. The patient was a former smoker and lived independently.

**Figure 1:**
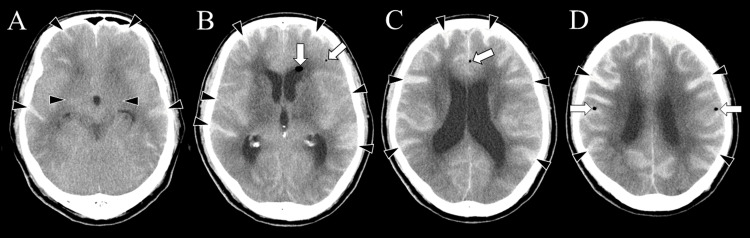
Initial non-contrast head CT at the referring hospital (Day 0, approximately 32 hours after myelography). (A–D) Axial CT images demonstrate hyperdense areas within the subarachnoid spaces along the cortical sulci (arrowheads), suggestive of subarachnoid hemorrhage. In addition, multiple low-density areas consistent with intracranial air are observed (arrows), including along the falx cerebri, within the anterior horn of the left lateral ventricle, and in the cortical subarachnoid space, suggestive of pneumocephalus.

On Day -1, spinal myelography was performed at the referring hospital as part of the preoperative evaluation for lumbar spine surgery. On Day 0, approximately 32 hours after myelography, a screening head CT scan was obtained at the referring hospital during routine follow-up. The CT scan demonstrated hyperdense areas within the cortical sulci of both cerebral hemispheres, raising suspicion for SAH (Figure 1). The patient was therefore referred to our hospital for further evaluation and was admitted emergently.

On arrival, the patient was alert with a Glasgow Coma Scale score of 15 (Eye opening: 4, Verbal response: 5, Motor response: 6). Vital signs were as follows: blood pressure 167/95 mmHg, heart rate 85 beats/min, respiratory rate 21 breaths/min, body temperature 36.5°C, and oxygen saturation 97% on room air. Neurological examination revealed no focal deficits, and no apparent head trauma was identified.

Laboratory evaluation showed no evidence of coagulopathy or bleeding tendency. The platelet count was 156 ×10³/μL, prothrombin time-international normalized ratio (PT-INR) was 1.01, activated partial thromboplastin time (APTT) was 25.8 seconds, and fibrinogen was 304 mg/dL. Inflammatory markers were within normal limits (C-reactive protein 0.12 mg/dL). D-dimer was mildly elevated at 2.82 μg/mL. Other biochemical parameters, including renal function and electrolytes, were unremarkable (Table 1).

**Table 1:** Laboratory findings on admission. PT-INR: Prothrombin time-international normalized ratio; APTT: Activated partial thromboplastin time; CRP: C-reactive protein; eGFR: Estimated glomerular filtration rate; AST: Aspartate Aminotransferase; ALT: Alanine Aminotransferase; HbA1C: Hemoglobin A1C; LDL-C: Low-density lipoprotein cholesterol

Parameter	Result	Reference range
WBC	5.73 ×10³/μL	3.5–9.0 ×10³/μL
Hemoglobin	13.7 g/dL	11.5–15.0 g/dL
Platelets	156 ×10³/μL	150–350 ×10³/μL
PT-INR	1.01	0.9–1.1
APTT	25.8 seconds	25–35 seconds
Fibrinogen	304 mg/dL	200–400 mg/dL
D-dimer	2.82 μg/mL	<1.0 μg/mL
Creatinine	1.05 mg/dL	0.6–1.1 mg/dL
eGFR	52.9 mL/min/1.73m²	>60 mL/min/1.73m²
AST	20 U/L	10–40 U/L
ALT	16 U/L	5–40 U/L
Glucose	149 mg/dL	70–140 mg/dL
HbA1c	5.90%	<6.0%
Triglycerides	239 mg/dL	<150 mg/dL
LDL-C	96 mg/dL	<140 mg/dL
CRP	0.12 mg/dL	<0.3 mg/dL
Sodium	141 mEq/L	135–145 mEq/L

Initial head CT demonstrated hyperdense areas along the cortical sulci of both cerebral hemispheres, suggestive of SAH (Figure 1). In addition, low-density areas compatible with intracranial air were observed along the falx cerebri and bilateral convexities, suggesting pneumocephalus. A similar low-density focus suspicious for intracranial air was also observed in the anterior horn of the left lateral ventricle. No skull fractures were identified.

**Figure 2:**
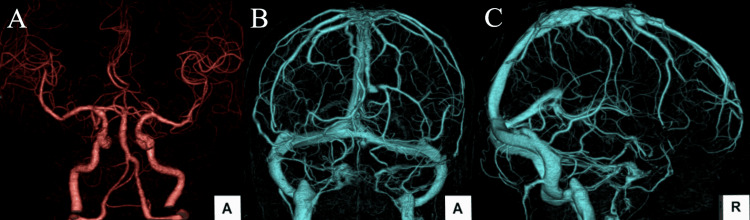
Three-dimensional CT angiography (CTA) and CT venography (CTV) demonstrating no vascular abnormalities. (A) Three-dimensional CTA shows no evidence of intracranial aneurysm, vascular malformation, or arterial dissection. (B) Frontal view of three-dimensional CTV demonstrates normal cerebral venous structures without evidence of venous sinus thrombosis. (C) Lateral view of three-dimensional CTV also shows no evidence of venous sinus thrombosis.

CT angiography and CT venography demonstrated no evidence of intracranial aneurysm, vascular malformation, arterial dissection, or cerebral venous sinus thrombosis (Figure 2).

Because the cause of SAH remained unclear, additional differential diagnoses, such as spinal dural arteriovenous fistula, were considered, and further investigations, including MRI and angiography, were planned. During the diagnostic process, the referring hospital was contacted for further clinical information, and it was confirmed that spinal myelography had been performed on the previous day.

**Figure 3:**
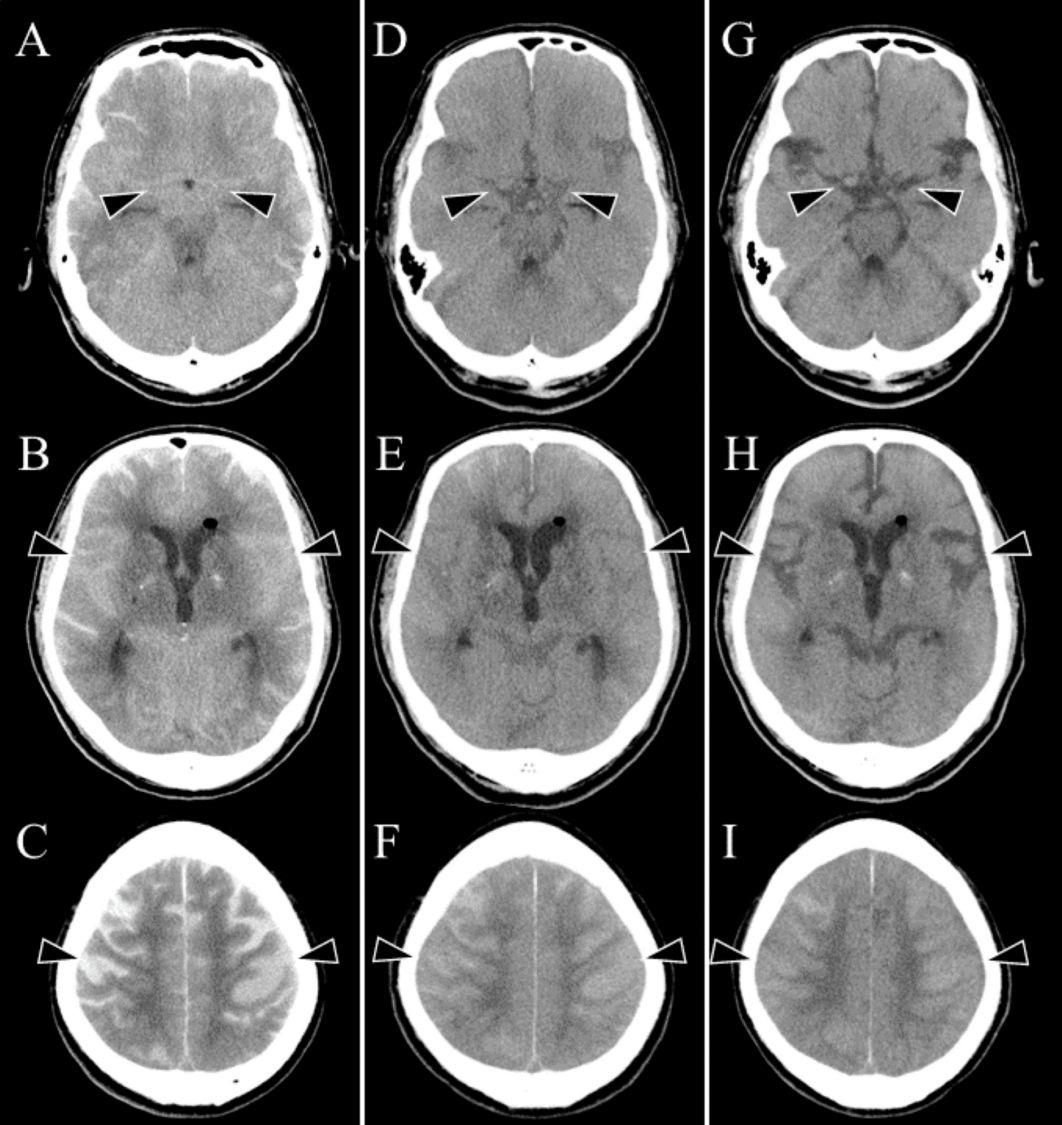
Serial non-contrast head CT demonstrating progressive decrease in subarachnoid hyperattenuation. (A–C) Initial CT obtained at the referring hospital (Day 0, approximately 32 hours after myelography) shows hyperdense areas within the subarachnoid spaces (arrowheads), mimicking subarachnoid hemorrhage. (D–F) Follow-up CT at our hospital (Day 1, approximately 40 hours after myelography) demonstrates a reduction in both the extent and attenuation of the hyperdense lesions (arrowheads). (G–I) Subsequent CT (Day 1, approximately 48 hours after myelography) shows further decrease in hyperattenuation (arrowheads).

**Figure 4:**
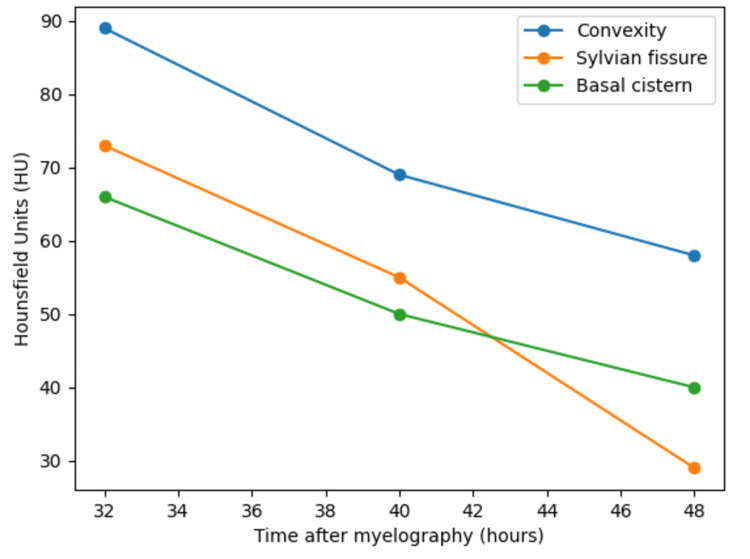
Temporal changes in CT attenuation values (Hounsfield units). Attenuation values measured in the convexity sulci, Sylvian fissure, and basal cistern demonstrated a progressive decrease over time following myelography, consistent with washout of intrathecal contrast material.

Follow-up CT examinations demonstrated a progressive decrease in the extent and attenuation of the hyperdense lesions (Figure 3). Hounsfield unit (HU) measurements were obtained retrospectively after multiple CT examinations. On the first CT obtained approximately 32 hours after myelography, the maximum attenuation values were 89 HU in the convexity sulci, 73 HU in the Sylvian fissure, and 66 HU in the basal cistern. On the subsequent CT performed approximately 40 hours after myelography, the attenuation values decreased to 69 HU, 55 HU, and 50 HU, respectively. A further CT performed approximately 48 hours after myelography demonstrated additional reductions to 58 HU, 29 HU, and 40 HU, respectively. These temporal changes corresponded with the progressive decrease in attenuation values shown in Figure 4 and strongly suggested washout of intrathecal contrast material rather than true hemorrhage.

The intracranial air observed on CT was also considered consistent with pneumocephalus related to the myelography procedure. Based on the history of recent spinal myelography, the absence of vascular abnormalities on CT angiography and venography, and the rapid temporal decrease in HU values on serial CT examinations, the imaging findings were diagnosed as pseudo-subarachnoid hemorrhage caused by migration of intrathecal contrast medium following myelography. The patient remained neurologically stable throughout hospitalization. Because no evidence of true intracranial hemorrhage was identified, the patient was managed conservatively and was discharged on Day 1.

## Discussion

Pseudo-SAH is an imaging phenomenon in which high attenuation within the subarachnoid spaces is observed on CT despite the absence of true hemorrhage. Although relatively uncommon, pseudo-SAH is an important diagnostic pitfall because it may be misinterpreted as aneurysmal subarachnoid hemorrhage, leading to unnecessary invasive investigations or inappropriate management.

Pseudo-SAH has been reported in a variety of clinical conditions, most commonly in association with diffuse cerebral edema. In conditions such as hypoxic-ischemic encephalopathy, severe traumatic brain injury, and encephalitis, marked brain swelling results in decreased attenuation of the brain parenchyma and narrowing of the subarachnoid spaces. This relative contrast enhancement of venous structures and compressed cerebrospinal fluid spaces produces an apparent hyperdensity mimicking SAH on CT [2].

In addition to these classical mechanisms, pseudo-SAH has increasingly been recognized following contrast media administration. Contrast-related pseudo-SAH is thought to result from leakage or redistribution of contrast material into the subarachnoid space, either through disruption of the blood-brain barrier or direct intrathecal migration. Several reports have described pseudo-SAH following neuroendovascular procedures, epidural interventions, and spinal myelography, in which iodinated contrast diffuses into the cerebrospinal fluid space and produces high attenuation mimicking hemorrhage [5-7]. In particular, intrathecal contrast migration after spinal procedures represents a well-defined mechanism, as demonstrated in previous case reports [6,7].

Differentiation between pseudo-SAH and true SAH is crucial in clinical practice. CT attenuation values provide an important clue: aneurysmal SAH typically demonstrates attenuation values of approximately 60-70 Hounsfield units (HU), whereas pseudo-SAH associated with diffuse cerebral edema often shows lower values, around 30-45 HU [2]. In contrast, contrast-related pseudo-SAH may demonstrate markedly elevated attenuation values, sometimes exceeding 100 HU, reflecting the high density of iodinated contrast material [5]. Therefore, unusually high attenuation within the subarachnoid space should raise suspicion for contrast-related pseudo-SAH.

Temporal changes in imaging findings also provide valuable diagnostic information. In contrast-related pseudo-SAH, hyperattenuation tends to decrease rapidly as the contrast agent is cleared, often within hours to a day. In contrast, true SAH typically persists for several days before gradual resorption. Follow-up CT is therefore a simple and effective tool for differentiation.

In the present case, the diagnosis was guided by the integration of clinical and radiological findings. The absence of typical symptoms of aneurysmal SAH, combined with the recent history of myelography, raised suspicion of a contrast-related phenomenon. In addition, the relatively high attenuation values and their rapid temporal decrease further supported the diagnosis of pseudo-SAH rather than true hemorrhage.

With the increasing use of contrast-enhanced diagnostic and therapeutic procedures, contrast-related pseudo-subarachnoid hemorrhage has been increasingly recognized in clinical practice, as reported in previous case reports and small case series [5-7]. Clinicians should therefore avoid relying solely on CT findings and instead integrate clinical history, attenuation values, temporal evolution, and additional imaging modalities.

Misdiagnosis of pseudo-SAH as true SAH may lead to unnecessary angiography or invasive treatments. This case highlights contrast-related pseudo-SAH as an important radiological pitfall and emphasizes the need for careful interpretation of CT findings in the appropriate clinical context.

## Conclusions

Contrast-related pseudo-subarachnoid hemorrhage is an important diagnostic pitfall that can closely mimic true subarachnoid hemorrhage on CT. Careful evaluation of clinical history, attenuation values, and temporal changes in imaging findings is essential for accurate diagnosis. Awareness of this condition is crucial to avoid unnecessary invasive investigations and inappropriate management.
